# Human annexin A5 promotes glioma progression by targeting the *MAPK/CD44* pathway

**DOI:** 10.3389/fonc.2025.1550961

**Published:** 2025-06-18

**Authors:** WeiXian Liu, Tao Xiong, Hu Sun, Ming Wang, JunGao Zhu, ChuanChuan Li

**Affiliations:** ^1^ Department of Neurosurgery, Zhejiang Hospital, Zhejiang, China; ^2^ Department of Orthopaedics, Zhejiang Hospital, Zhejiang, China; ^3^ Department of Thoracic surgery, Zhejiang Hospital, Zhejiang, China

**Keywords:** glioma, anxa5, CD44, MAPK, gene expression omnibus

## Abstract

**Background:**

Gliomas are the most common intracranial malignant tumors. In this study, we aimed to identify the hub genes and investigate the pathophysiological significance of *ANXA5* in glioma.

**Methods:**

The differentially expressed genes (DEGs) between tumor and adjacent tissues from glioma patients were acquired from the Gene Expression Omnibus (GEO) database. Functional enrichment analysis and protein-protein interaction (PPI) network construction of overlapping DEGs were performed. The GEPIA and CGGA databases were used to explore hub gene expression. The effect of hub genes on prognosis and tumor-infiltrating immune cells was analyzed via GEPIA, CGGA, and TIMER2 databases. Additionally, *ANXA5* expression was measured by qRT-PCR and Western blotting. The effects of *ANXA5* were assessed by CCK-8, colony formation, Transwell, and flow cytometry assays. Moreover, the roles of *ANXA5* were identified *in vivo*.

**Results:**

The DEGs were enriched in cell surface receptor signaling pathway, immune response, and *MAPK* signaling pathway. The selected hub genes were included *ANXA5*, *STAT1*, *CD44*, *CAV1*, *ANXA2*, and *MAPT*. Among them, expression of *ANXA5*, *STAT1*, *CD44*, *CAV1*, and *ANXA2* was strongly correlated with patient prognosis and was also involved in the tumor microenvironment. Furthermore, *ANXA5* knockdown significantly inhibited the migration and proliferation of glioma cells *in vitro* and *in vivo*. Meanwhile, we found that the expression of *CD44* was monitored by *ANXA5*, and *ANXA5* promoted the migration and proliferation of glioma cells via the *MAPK/CD44* pathway.

**Conclusion:**

Taken together, our data showed that *ANXA5* could contribute to cell proliferation and metastasis of glioma by targeting the *MAPK/CD44* axis.

## Introduction

Gliomas are the most common primary intracranial malignant tumors in adults, Patients diagnosed with GBM typically have an average survival time of 14 months (1), with merely 2% of them living for three years post-surgery (2). Current treatments for gliomas include a combination of surgical resection, radiotherapy, and chemotherapy (3, 4). Although much progress has been made, patient survival is still unsatisfactory due to the high metastatic ability of gliomas (5). In the 2021 World Health Organization (WHO) Central Nervous System (CNS) tumor classification, low-grade glioma (LGG) includes CNS WHO classification 1-2, and high-grade glioma (HGG) includes CNS WHO classification 3-4, and LGG accounts for 6% of primary tumors in the adult CNS and has a restricted efficacy of immunotherapy (6, 7). Therefore, there is an urgent need for new approaches to effectively treat gliomas and significantly reduce mortality and further research into their underlying molecular mechanisms.

In this study, we first investigated the GEO datasets, GSE12657, GSE42656, GSE50161, and GSE209547, carrying high-throughput sequencing data human glioma samples to identify the key prognostic biomarkers or the potential targets in glioma. The protein-protein interaction (PPI) network analysis showed that *ANXA5*, *STAT1*, *CD44*, *CAV1*, *ANXA2*, and *MAPT* may serve as candidate biomarkers in glioma diagnosis. Among them, Human annexin A5 (*ANXA5*) is an important functional protein molecule of the annexin family in the human body and may related to recurrent pregnancy loss (8). hANXA5 has various functions such as antiphospholipase, anticoagulant, antikinase, Ca^2+^ channel activity, and phosphatidylserine binding (9). Currently, many studies also have found that hANXA5 plays an important role in a variety of tumors. For example, Su et al. found that the increased *ANXA5* expression was markedly related to the poor prognosis and a high level of immune cell infiltration in stomach adenocarcinoma (10). Gounou et al. demonstrated that down-regulation of *ANXA5* and *ANXA6* blocks membrane repair in triple-negative breast cancer cell lines during metastasis (11). Moreover, Yang et al. reported that *ANXA5* overexpression contributes to glucocorticoid resistance in B-cell acute lymphoblastic leukemia (12). In contrast, Wang et al. expounded that the inhibition of *ANXA5* resulted in an increase in proliferation, migration, invasion, and a decrease in apoptosis of HeLa cells by activating the *PI3K/Akt* pathway (13). Generally, the aforementioned studies suggest that *ANXA5* may still be a potential target for tumor-specific therapeutic and diagnostic strategies for explaining cancer development and progression. However, the expression, biological roles, and potential molecular mechanisms of *ANXA5* in gliomas are still unclear. Hence, the roles of *ANXA5* on the proliferation, migration, invasion, cell cycle, and apoptosis of glioma cells as well as its relevant mechanism were investigated.

More importantly, *ANXA5* was positively correlated with Cluster of differentiation-44 (*CD44*) according to the Chinese Glioma Genome Atlas (CGGA) database. *CD44* is a transmembrane glycoprotein involved in signal transduction between cells and the extracellular matrix and is associated with the invasion, metastasis, and drug resistance as well as the formation of tumor microenvironment in cancers (14, 15). As we know, *CD44* promotes the progression of cancer by activating crucial signaling pathways. For example, a study by Ji et al. revealed that *CD44* helps in the migration capacity of U-2 OS and MG-63 cells through the *Wnt/β-catenin* pathway (16). Similarly, Du et al. found that overexpression of *CD44* promoted cell proliferation, migration, and invasion in clear cell renal cell carcinoma cells through the activation of the *HAS1/MMP9* pathway (17). More importantly, the intratumor activation of the mitogen-activated protein kinases (MAPKs) and the *PI3K*/protein kinase B (*AKT*) pathways participate in the role and mechanism of the *CD44* in the glioblastoma tissue (18). The MAPKs include extracellular signal-regulated kinase (*ERK*), *p38*, and c-Jun NH(2)-terminal kinase (*JNK*) pathways that are activated by diverse extracellular and intracellular signals to mediate the malignant characteristics in many malignant tumors (19, 20). Further study on the effects of *ANXA5* may contribute to understanding the potential mechanism associated with *MAPK/CD44* pathway in glioma progression.

As a result, we confirmed the up-regulation of *ANXA5* in glioma cell lines, and further elucidated its biological roles and underlying molecular mechanisms *in vivo* and *in vitro*, which may provide novel targets for personalized diagnosis and treatment of glioma.

## Materials and methods

### Data collection from GEO database and identification of differentially expressed genes

Raw data from GSE12657 data set containing 7 glioma samples and 5 paired neighboring noncancerous tissues, GSE42656 data set containing 5 glioma samples and 8 paracarcinoma tissues, GSE50161 data set containing 117 glioma samples and 13 paracarcinoma tissues, and GSE209547 data set containing 5 glioma samples and 5 paracarcinoma tissues was downloaded from the National Center for Biotechnology Information (NCBI) Gene Expression Omnibus (GEO) database (https://www.ncbi.nlm.nih.gov/geo) with the keywords “glioma” and “homo sapiens”. Next, we performed differential expression analysis with the GEO2R online tool (https://www.ncbi.nlm.nih.gov/geo/geo2r/) to identify the differentially expressed genes (DEGs), according to the |log FC (Fold chang)| (>1) and *P* (<0.05) value, respectively. Finally, a Venn diagram was drawn using Bioinformatics & Evolutionary Genomics (http://bioinformatics.psb.ugent.be/webtools/Venn/) to select the overlapping DEGs from the result of the intersection of four datasets.

### PPI network analysis

The Search Tool the Retrieval of Interacting Genes (STRING) database (http://www.string-db.org) was applied to construct the PPI network of overlapping DEGs. Additionally, the obtained interaction network was imported into Cytoscape (version 3.7.2) software to screen core modules. And then, we used the CytohHubba plug-in in Cytoscape software to identify hub genes in the core subnetworks according to the MCC, Degree, and EPC algorithm.

### Functional enrichment analysis of overlapping DEGs and verification of hub genes

Functional enrichment analysis for Gene Ontology (GO) terms includes three-term groups: biological processes (BP), molecular functions (MF), and cellular components (CC) were performed by The Database for Annotation, Visualization and Integrated Discovery (DAVID, https://david.ncifcrf.gov/). In addition, the Kyoto Encyclopedia of Genes and Genomes (KEGG) pathway analysis was used to illustrate the significant pathways of overlapping DEGs. Next, the expression levels of core genes were analyzed by using the GEPIA website (http://gepia.cancer-pku.cn/). Furthermore, the prognostic significance of hub genes in glioblastoma multiforme (GBM) and LGG patients was investigated on the GEPIA platform. For further external validation of hub genes, the mRNA expression level in different WHO classifications and prognostic value of hub genes in primary glioma were explored using the CGGA database (http://www.cgga.org.cn/). We also estimated the relationship between the expression level of *ANXA5* and the rest of the hub genes in the CGGA database. To verify the prognostic value of the hub genes, receiver operating characteristics (ROC) were analyzed using the MedCalc software (v15.2.1) in the GSE50161 dataset.

### Cell culture

Glioma cell lines T98G, U251, luciferase-transfected U251-Luc, U373, A172, SHG44, U118, LN382, and the human normal glial cell line HEB were purchased from iCell Bioscience Inc (Shanghai, China). All cells were maintained in Dulbecco’s modified Eagle’s medium (DMEM, Transgen Biotech Co., LTD, Beijing, China) supplemented with 10% fetal bovine serum (FBS, Zhejiang Tianhang Biotechnology Co., LTD, China), 100 U/ml penicillin, and 100 µg/ml streptomycin at 37°C in a 5% CO_2_ incubator.

### Cell transfection

To establish the stably transfected cells overexpressing *ANXA5*, the wildtype *ANXA5* sequence was amplified using PCR, then sub-cloned into plasmid pcDNA3.1 (Invitrogen, USA). In *ANXA5* and *CD44* knockdown model, shRNA sequences against *ANXA5* (shANXA5#1/shANXA5#2) and small interfering (si) RNA sequences against *CD44* (si_CD44) or negative control (shCtrl/si_CD44_NC) were constructed or synthesized by Genepharm Co. Ltd. (Shanghai, China). Following annealing, shRNAs were introduced in the lentiviral pU6-Luc-Puro vector (Genepharm Co. Ltd.). U251 and SHG44 cells (2×10^5^ cells/well) were plated in 6-well plates at 24 h and then transfected with shRNA lentiviral for *ANXA5*, the overexpression plasmid pcDNA3.1-ANXA5 (oe-ANXA5) or si_CD44 at 80% confluence using Lipofectamine 2000 (Thermo Fisher Scientific) based on the manufacturer’s protocol. After 48 h of transfection, the transfected efficiency was estimated with reverse transcription-quantitative polymerase chain reaction (RT-qPCR) and Western blot assays.

### Cell proliferation assay

U251 and SHG44 cells transfected with either shANXA5#1 and shANXA5#2 or shCtrl were seeded into 96-well plates at a density of 100 μl 3×10^3^ cells per well and measured after 24, 48, 72 and 96 h. At the specific time point, 10 μl of Cell Counting Kit-8 (CCK-8) solution was added to each well for incubation with the transfected cells for 2 h. After that, the optical density at a wavelength of 450 nm was measured using a microplate reader (Molecular Devices). For colony formation assays, the transfected U251 and SHG44 cells were plated into 6-well plates (2,000 cells/well) and incubated for 14 days. Next, the cells were fixed with 4% paraformaldehyde for 15 min and stained with 1% crystal violet (Sigma-Aldrich) for 10 min. Finally, the images were captured using a light microscope (Olympus, Tokyo, Japan) and quantified with Image J (National Institutes of Health).

### Cell migration and invasion assays

Cell migration assays were performed using uncoated 24-well Transwell chambers (8 µm pores; BD Biosciences). And, the 24-well Transwell chambers were pre-coated with Matrigel (BD Biosciences) for invasion assays. U251 and SHG44 cells transfected with either shANXA5#1, shANXA5#2, oe-ANXA5, si_CD44 alone or in combination (4x10^4^ cell/well) suspended in 150 µl DMEM (with 1% FBS) were added into the top chamber. Then, 600 μL of complete medium with 20% FBS was added into the lower chamber. Following that, the cells were incubated at 37°C for 48h. Subsequently, the Transwell chambers were fixed with 4% paraformaldehyde for 20 min, followed by staining with 1% crystal violet (Solarbio, Beijing, China) for 10 min. Then, the stained cells were counted under an inverted microscope and counted.

### Flow cytometry assays

Cell apoptosis rate and cell cycle distribution of transfected U251 and SHG44 cells were measured using flow cytometry. For cell apoptosis assays, the treated U251 and SHG44 cells were trypsinized, washed, and then stained with 5 μL of FITC-conjugated anti-Annexin V antibody and 10 μL of propidium iodide (PI) for 15 min out of direct sunlight, followed by measuring with a NovoCyte flow cytometer (Agilent Technologies). For cell cycle assays, the transfected U251 and SHG44 cells were fixed with ethanol and then stained with PI. Immediately afterward, the cell cycle distribution was assessed by the flow cytometer. To observe the expression of *CD44* in transfected U251 and SHG44 cells, an APC-conjugated anti-human/mouse *CD44* antibody (F1104403, MULTISCIENCES, Hangzhou, China) was utilized to stain the cells. After digestion and centrifugation, the cells were resuspended in 200 μL of PBS and stained with 5 μl of *CD44* antibody for 30 min at 4°C. then followed by an investigation by applying flow cytometry.

### *In vivo* assays

Four- to five-week-old female BALB/c nude mice (16~18 g) were purchased from the SLAC Laboratory Animal Co., Ltd [SCXK(Hu)2022-0004, Shanghai, China] and the study was approved by Institutional Animal Care and Use Committee of Zhejiang Eyong Pharmaceutical Research and Development Center (NO. ZJEY-20221107-03). All mice were housed in a temperature- and humidity-controlled rooms with a 12/12 h light/dark cycle. For orthotopic xenografts assays, gGuided intracranial injections using stereotactic frame (Leica, Germany) in nude mice were conducted by delivering 1×10^5^ of U251-Luc treated with shANXA5 lentiviral vector. Cells were resuspended in 5 μL PBS and injection were made into the striatum using a microsyringe. The orthotopic tumor sizes were estimated every 7 days using an AniView 100 *In vivo* imaging analyzer (Guangzhou Biolight Biotechnology Co., Ltd, China). Bioluminescent imaging data are shown as (photons/sec/cm2/steradian). For *in vivo* xenograft experiments, 2×10^6^ transfected SHG44 cells were injected into nude mice subcutaneously. Tumor volume was monitored every 5 days and calculated using the formula: volume (mm^3^) = width^2^×length/2. The mice were sacrificed after 30 days, and all the tumor tissues were surgically collected and weighed. Subsequently, the brain and tumor tissues were observed by hematoxylin and eosin (H&E) staining and Immunohistochemistry (IHC) assays.

### HE staining and IHC analysis

The brain tissues from the *in situ* tumor model and the subcutaneous xenotransplanted tumor tissues were fixed with 4% paraformaldehyde, embedded in paraffin, and sectioned into 4 µm sections. Next, the sections were dewaxed, rehydrated, and stained with hematoxylin-eosin (HE). For IHC assays, the paraffin-embedded tissue slides were incubated with primary antibodies (*ANXA5*, DF6580, Affinity; *Ki67*, AF0198, Affinity; *CD44*, DF6392, Affinity) overnight at 4 °C, followed by incubation with anti-rabbit IgG-HRP (ab97080, Abcam) antibody at room temperature for 30 min. Subsequently, the sections were stained with 3,3’-diaminobenzidine (DAB) solution (Beyotime, China) for 5 min. Finally, the images were taken by a DS-U3 microscopy (Nikon, Tokyo, Japan).

### RT-qPCR assays

Total RNA from glioma cell lines was extracted with EZ-10 DNAaway RNA Mini-prep Kit (Sangon Biotech (Shanghai) Co., Ltd. China) and cDNA was synthesized using a HiFiScript cDNA Synthesis Kit (Jiangsu Cowin Biotech Co., Ltd, China) according to the manufacturer’s instructions. Next, the qPCR was performed with the SYBR Green qPCR Master Mix Kit (Yeasen Biotechnology (Shanghai) Co., Ltd. China) on a LightCycler^®^ 96 real-time PCR system (Roche, Basel, Switzerland). β-actin was used as an internal control for *ANXA5*. The primers used: Human *ANXA5* (NM_001154.4) F: 5’-AGCGGGCTGATGCAGAAAC-3’, R: 5’-ACTTCGGGATGTCAACAGAGT-3’; *β-actin* F: 5’-CATGTACGTTGCTATCCAGGC-3’, R: 5’-CTCCTTAATGTCACGCACGAT -3’ were designed and synthesized by Sangon Biotech (Shanghai, China), and the relative expression levels were calculated using the 2^-ΔΔCt^ method.

### Western blot assays

Total protein was extracted from transfected U251 and SHG44 cells using RIPA lysate (Beyotime, China), and the protein concentration was measured with a bicinchoninic acid (BCA) protein assay kit (Beyotime). Then, a total of 30 µg protein samples were separated by sodium dodecyl sulfate-polyacrylamide gel electrophoresis (SDS-PAGE) and further transferred on PVDF membranes (Millipore, USA). After that, the PVDF membrane was blocked with 5% non-fat milk at 37°C for 1 h, and then incubated with the primary antibodies: Anti-*ANXA5* (1:1000, DF6580, Affinity), anti-*Bax* (1:1000, AF0120, Affinity), anti-*Bcl-2* (1:1000, AF6139, Affinity), anti-*caspase-3* (1:1000, AF6311, Affinity), anti-*MMP-2* (1:1000, 66366-1-Ig, proteintech), anti-*MMP-9* (1:1000, ab283575, Abcam), anti-*vimentin* (1:1000, ab20346, Abcam), anti-*E-cadherin* (1:1000, 20874-1-AP, proteintech), anti-*p21* (1:1000, AF6290, Affinity), anti-*cyclin A* (1:1000, AF0142, Affinity), anti-*CDK1* (1:1000, ab133327, Abcam), anti-*cyclin B* (1:1000, AF6168, Affinity), anti-*CDK2* (1:1000, AF6237, Affinity), anti-*CD44* (1:1000, DF6392, Affinity), anti-p-*P38* (1:1000, AF4001, Affinity), anti-*P38* (1:1000, AF6456, Affinity), anti-p-*JNK* (1:1000, AF3318, Affinity), anti-*JNK* (1:1000, AF6319, Affinity), anti-p-*ERK1/2* (1:1000, 4370T, Cell Signaling Technology), anti-*ERK1/2* (1:1000, 4695T, Cell Signaling Technology), and anti-β-actin (1:5000, AF7018, Affinity) overnight at 4°C. Next day, the membranes were washed with TBST and incubated with secondary antibodies conjugated with HRP: anti-rabbit IgG (1:6000; #7074; Cell Signaling Technology) or anti-mouse (1:6000; #7076; Cell Signaling Technology) for 2 h at room temperature. Finally, the protein bands were visualized with an enhanced chemiluminescence (ECL) kit and quantified using Image J (NIH, Bethesda, Maryland, USA).

### Statistical analysis

All statistical data were represented as means ± standard deviation (SD). Differences between the two different groups were estimated by a Two-tailed Student’s *t-tes*t. The differences between multiple groups of results were measured with a one-way analysis of variance, and *P < 0.05* was considered statistically significant. All experiments were performed at least three times.

## Results

### Identification of overlapping DEGs in glioma

**Figure 1:**
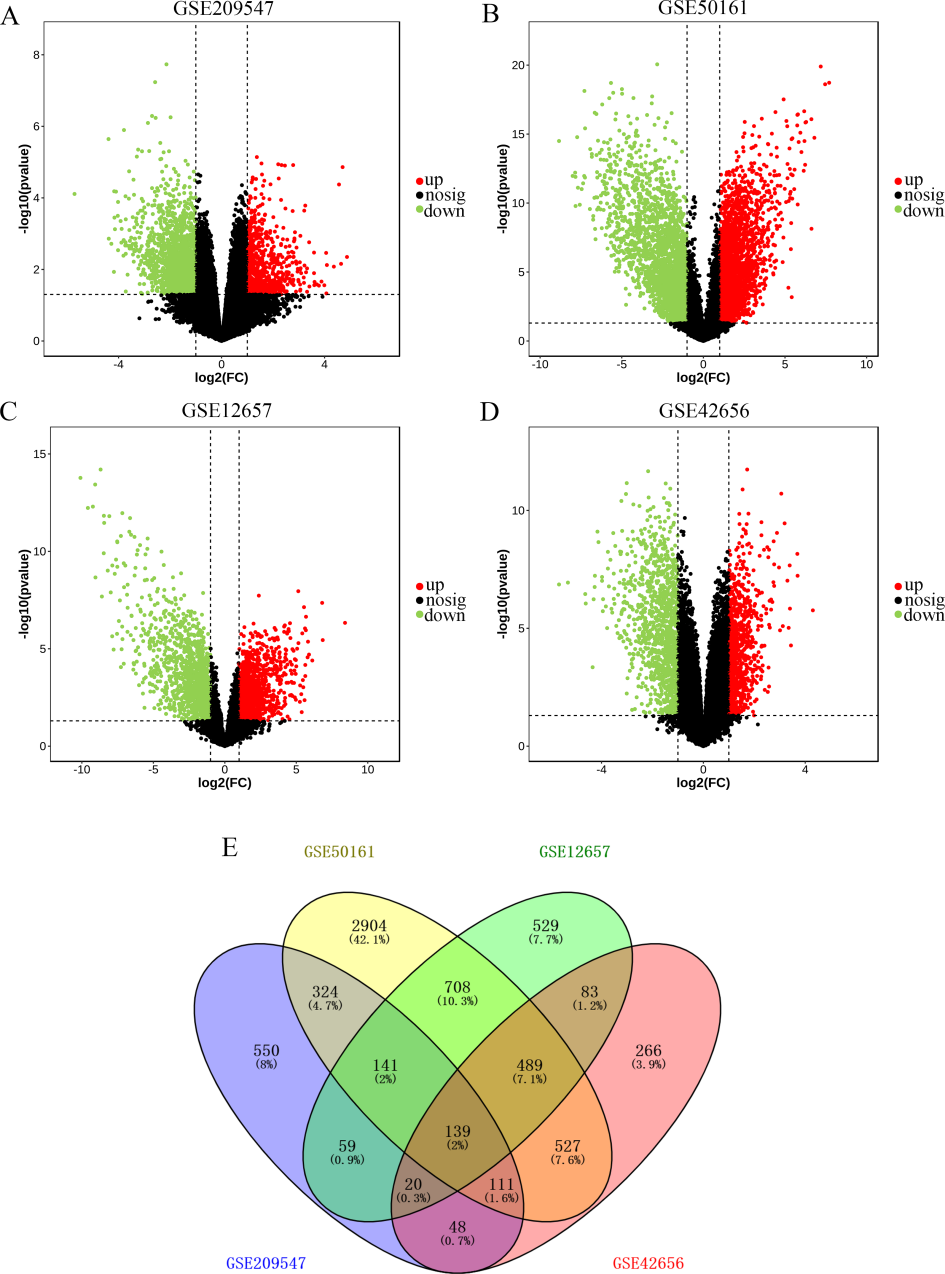
Identification of differentially expressed genes (DEGs) in four GEO datasets. Volcano plot of DEGs in GSE209547 **(A)**, GSE50161 **(B)**, GSE12657 **(C)**, and GSE42656 **(D)** datasets in GEO database. Red represents up-regulated DEGs and green represents down-regulated DEGs. **(E)** Venn diagram to identify overlapping DEGs from the four datasets.

To identify glioma-associated DEGs, we first identified the DEGs in the GEO database. The profiles of GSE209547, GSE50161, GSE12657, and GSE42656 were independently analyzed by the online software GEO2R to filter the DEGs between glioma samples and control samples. Considering |log_2_^FC^|> 1 and *P-value* < 0.05 as the cutoff condition, the DEGs in GSE209547, GSE50161, GSE12657, and GSE42656 were screened and illustrated in **Figures 1A–D**. Besides, we found 139 differentially expressed overlapping glioma-associated DEGs for further analysis (**Figure 1E**).

### PPI network construction and module analysis

**Figure 2:**
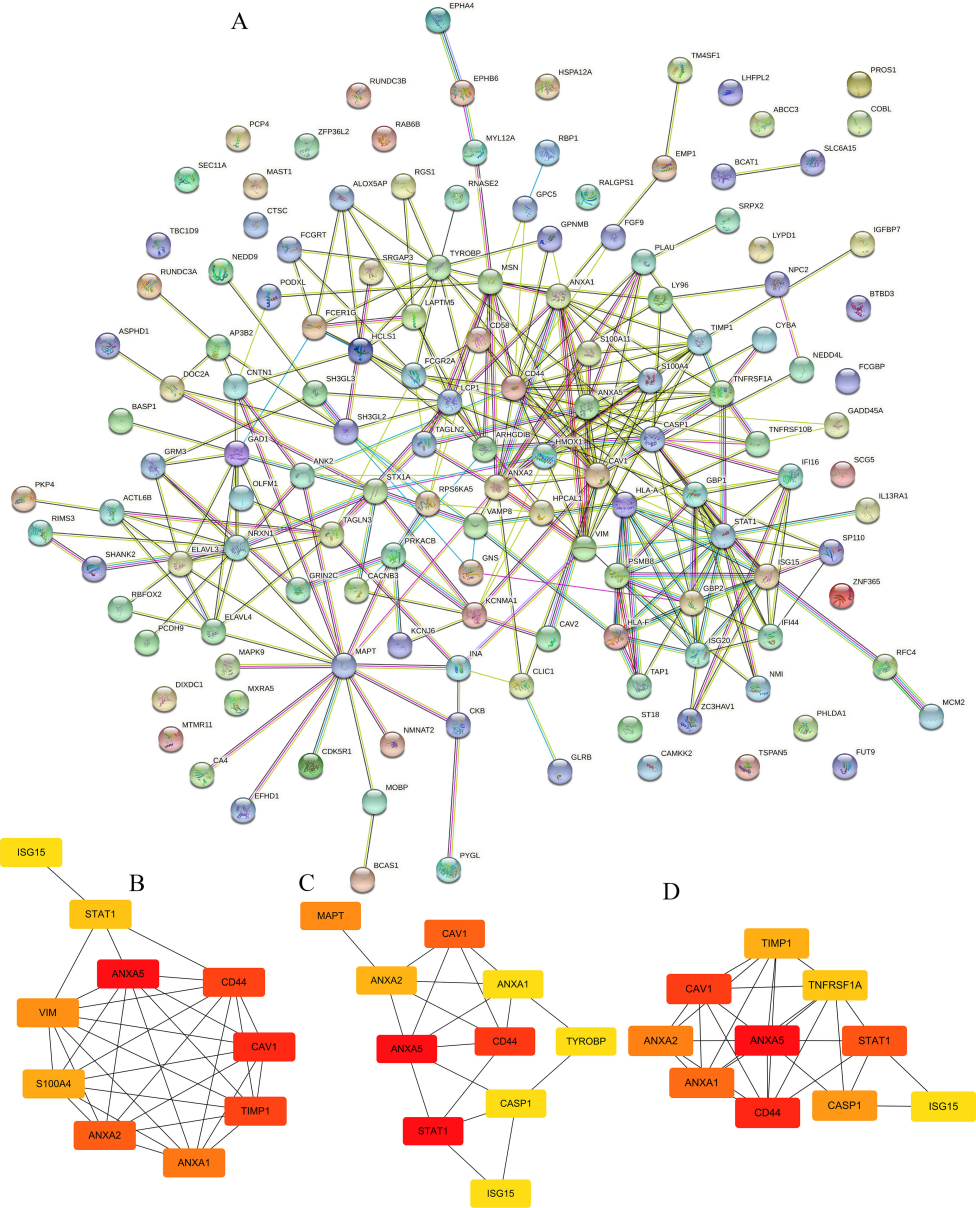
Protein-protein interaction (PPI) network analysis of DEGs and hub genes identification. **(A)** PPI network of the DEGs was conducted using STRING database (https://cn.string-db.org/). The hub genes depended on **(B)** MMC, **(C)** Degree and **(D)** EPC algorithms in CytoHubba, a plugin of Cytoscape. At last, the top 6 hub genes in different algorithms were used for further exploration.

The overlapping DEGs were imported into the STRING database to construct the PPI network to reveal its interaction relationships. As a result, we found a total of 110 nodes and 294 edges in the PPI network by using Cytoscape software (**Figure 2A**). CytoHubba contained several algorithms, and the three significant modules using MMC, Degree, and EPC algorithm were shown in **Figures 2B–D, respectively**. Finally, six genes (*ANXA5*, *STAT1*, *CD44*, *CAV1*, *MAPT*, and *ANXA2*) with the highest degree values were selected as the hub genes for glioma by using the cytoHubba plugin for expression level and prognosis analyses.

### Functional enrichment analysis of overlapping DEGs and prognostic analyses of hub genes in glioma

**Figure 3:**
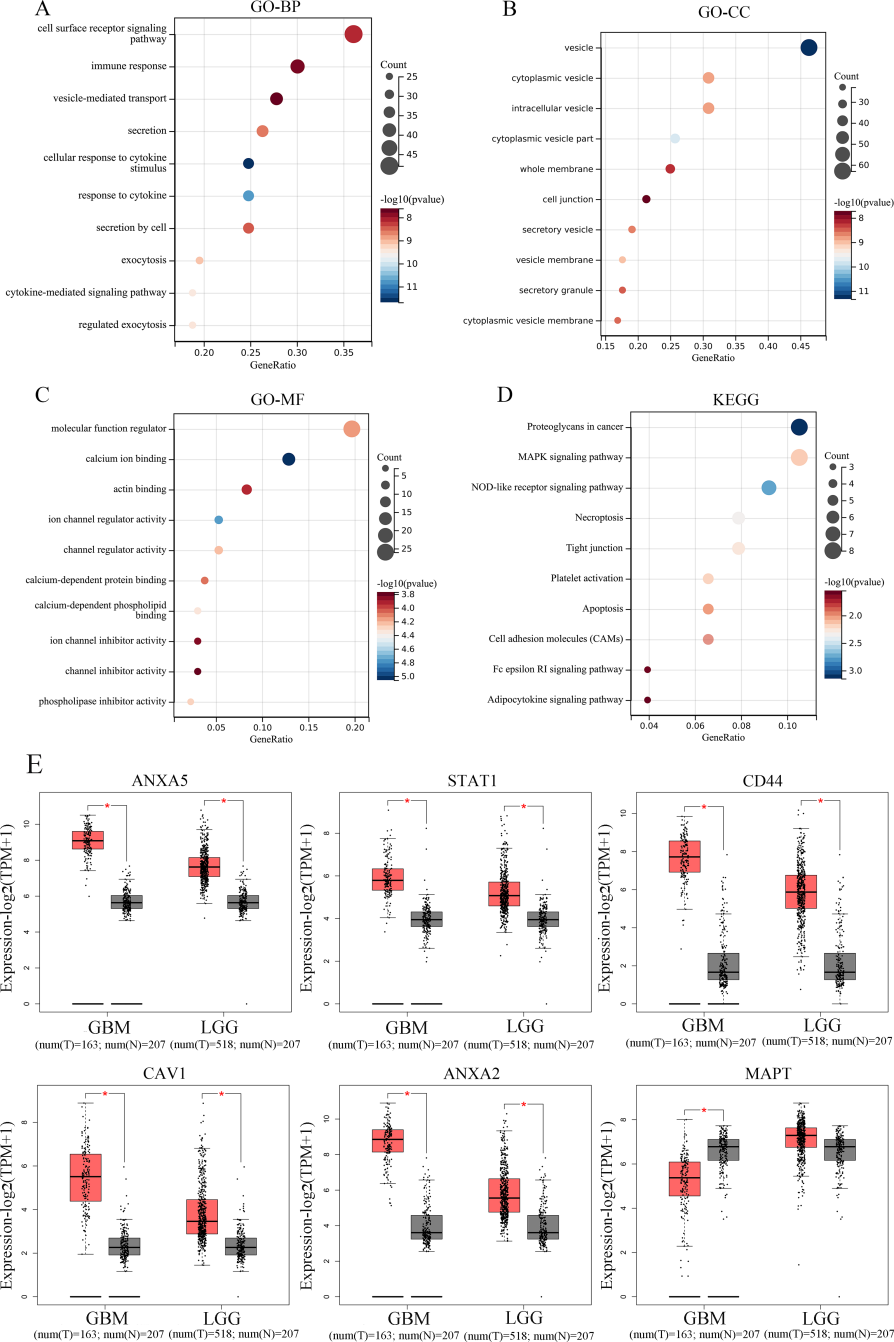
Functional enrichment analysis and expression levels of DEGs. **(A)** The top 10 significantly enriched GO **(A)** BP, **(B)** CC and **(C)** MF terms as well **(D)** KEGG pathways of DEGs in glioma. GO, gene ontology; KEGG, Kyoto encyclopedia of genes and genomes; BP, Biological process; CC, Cellular component; MF, Molecular function. The expression level of top 6 hub genes according to TCGA database. **(E)** The expression levels of top 6 hub genes, ANXA5, STAT1, CD44, CAV1, ANXA2, and MAPT from the GEPIA database in LGG and GBM. The red box plots represent tumor samples, and gray box plots represent normal samples.

**Figure 4:**
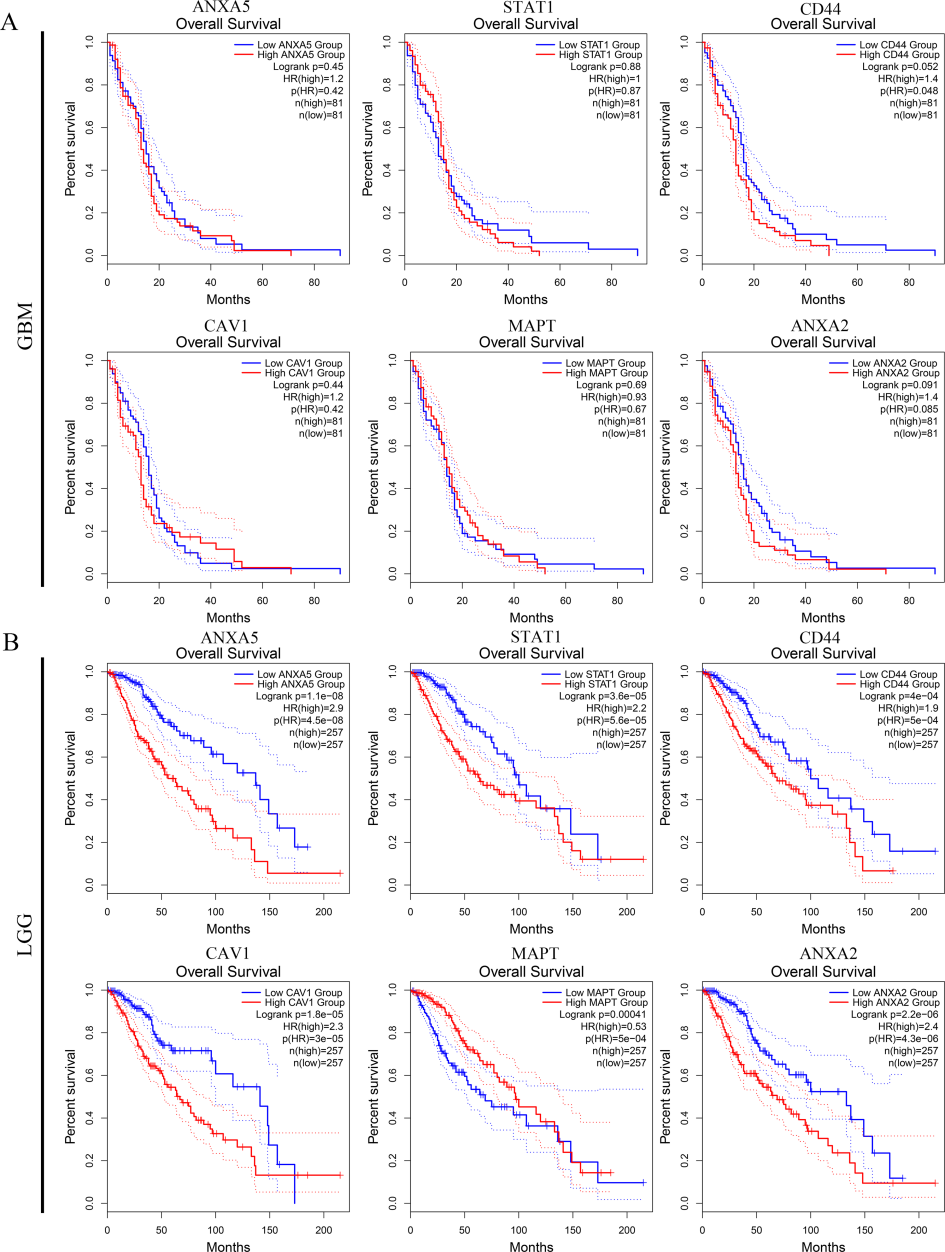
Prognostic value of top 6 hub genes. The survival curves of continuous variables of ANXA5, STAT1, CD44, CAV1, MAPT, and ANXA2 in GBM **(A)** and LGG **(B)** according to the GEPIA database.

**Figure 5:**
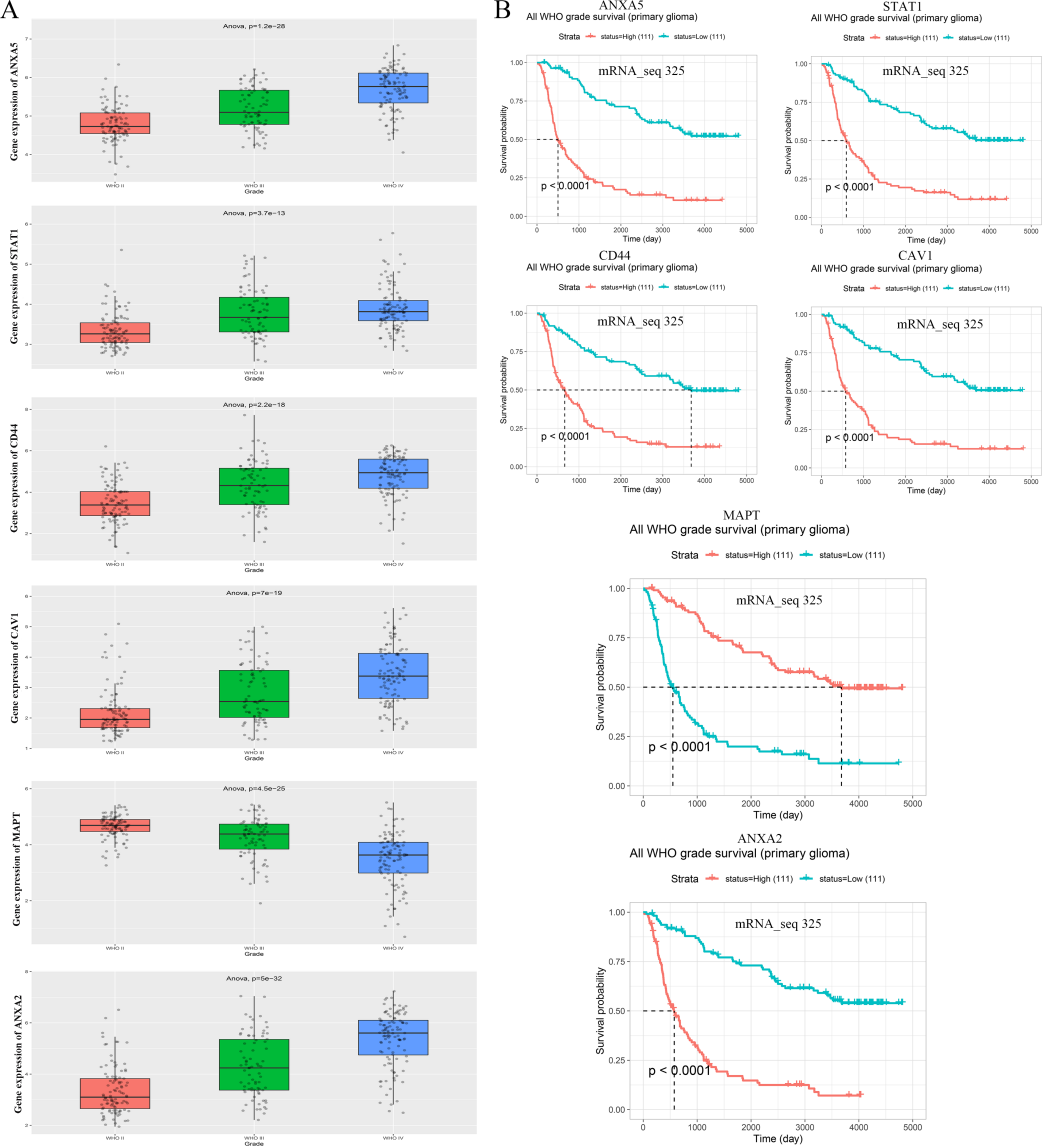
Validation of the expression level and clinical significance of top 6 hub genes. **(A)** The correlation of ANXA5, STAT1, CD44, CAV1, MAPT, and ANXA2 expression level with WHO grade and **(B)** survival analysis of the top 6 hub genes from CGGA database.

**Figure 6:**
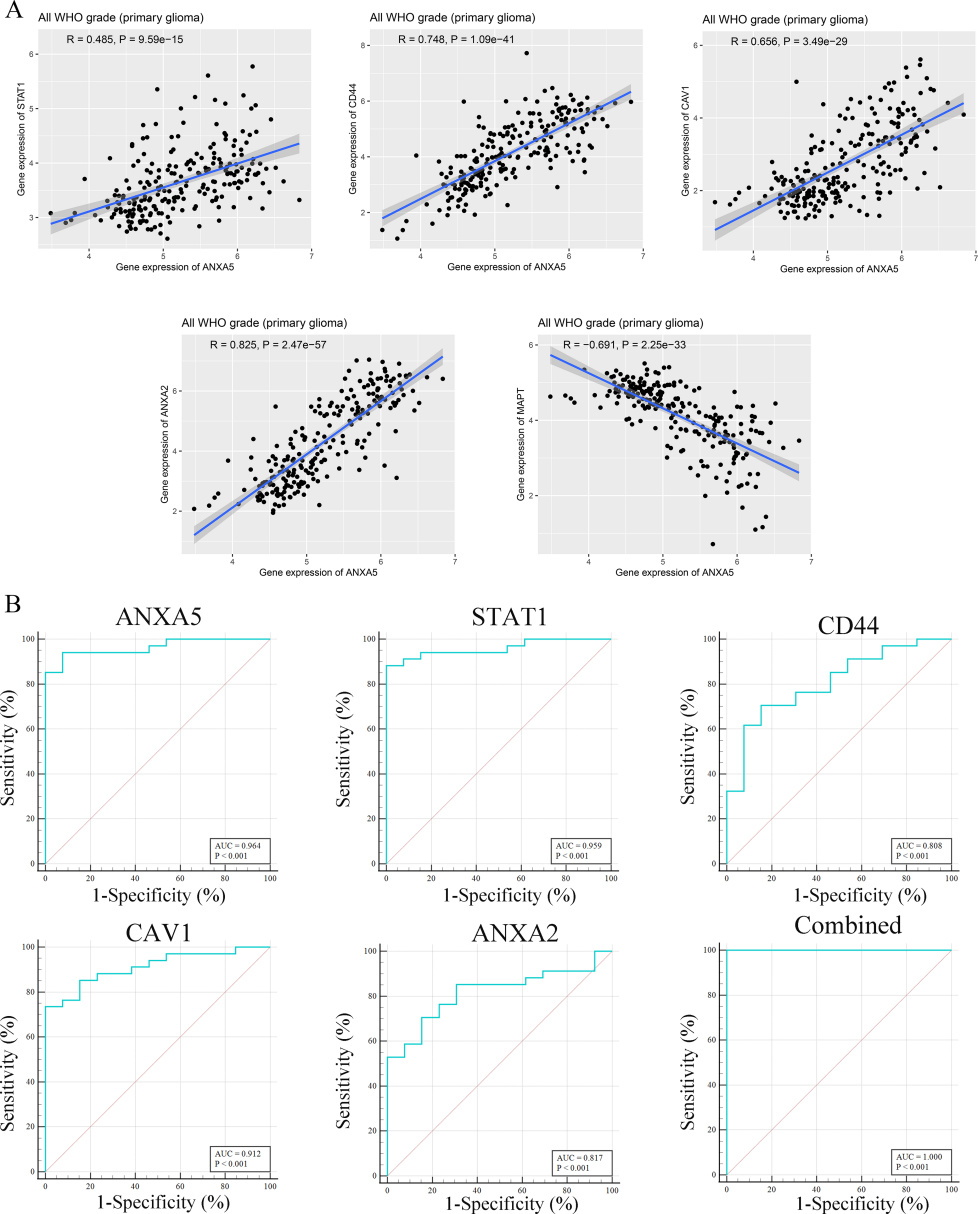
The interaction and diagnosis value of top 6 hub genes. **(A)** The interaction between ANXA5 and STAT1, CD44, CAV1, MAPT, and ANXA2 expressions, as demonstrated by CGGA database. **(B)** ROC analysis of ANXA5, STAT1, CD44, CAV1, and ANXA2 genes in GSE50161 dataset.

A total of 139 overlapping DEGs were analyzed by the DAVID platform. The top 10 significant terms from the GO enrichment analysis showed that in the BP category, the overlapping DEGs were associated with cell surface receptor signaling pathway, immune response, vesicle-mediated transport, and cellular response to cytokine stimulus (**Figure 3A**). For the CC category, the overlapping DEGs were involved in cytoplasmic vesicle, intracellular vesicle, and whole membrane (**Figure 3B**). For the MF category, the overlapping DEGs were enriched in molecular function regulator, calcium ion binding, actin binding, and ion channel regulator activity (**Figure 3C**). Furthermore, the KEGG pathway analysis showed that the overlapping DEGs were enriched in proteoglycans in cancer, *MAPK* signaling pathway, and NOD-like receptor signaling pathway (**Figure 3D**). Furthermore, the expression levels and overall survival analysis of hub genes were also investigated in the GEPIA online tool. The results showed that *ANXA5*, *STAT1*, *CD44*, *CAV1*, and *ANXA2* not only were upregulated in GBM and LGG tissue samples (**Figure 3E**) but also were significantly associated with worse prognosis in LGG patients, particularly the *ANXA5*, while no significant difference was found in GBM patients (**Figures 4A, B**). However, low expression of *MAPT* was observed in GBM patients and was positively related to the poor overall survival of LGG patients. Additionally, we further used the CGGA database to validate expression levels and prognostic value of hub genes. Similar results were found in the CGGA database (**Figures 5A, B**). Moreover, we found that the gene expression of *ANXA5* was positively associated with the expression of *STAT1*, *CD44*, *CAV1*, and *ANXA2*, while was negatively correlated with *MAPT* gene expression (**Figure 6A**). More importantly, the ROC curves were adopted to estimate the diagnostic value of hub gene expression to distinguish glioma tissues from normal tissues. *ANXA5* (AUC: 0.964), *STAT1* (AUC: 0.959), *CD44* (AUC: 0.808), *CAV1* (AUC: 0.912), *ANXA2* (AUC: 0.817), and combined (AUC:1.000) were found in the GSE50161 dataset (**Figure 6B**). Generally, these results suggested that *ANXA5*, *STAT1*, *CD44*, *CAV1*, *MAPT*, and *ANXA2* might play a critical role in the pathogenesis of glioma, suggesting that the hub genes might be a promising biomarker of glioma.

### Immune infiltration analysis

**Figure 7:**
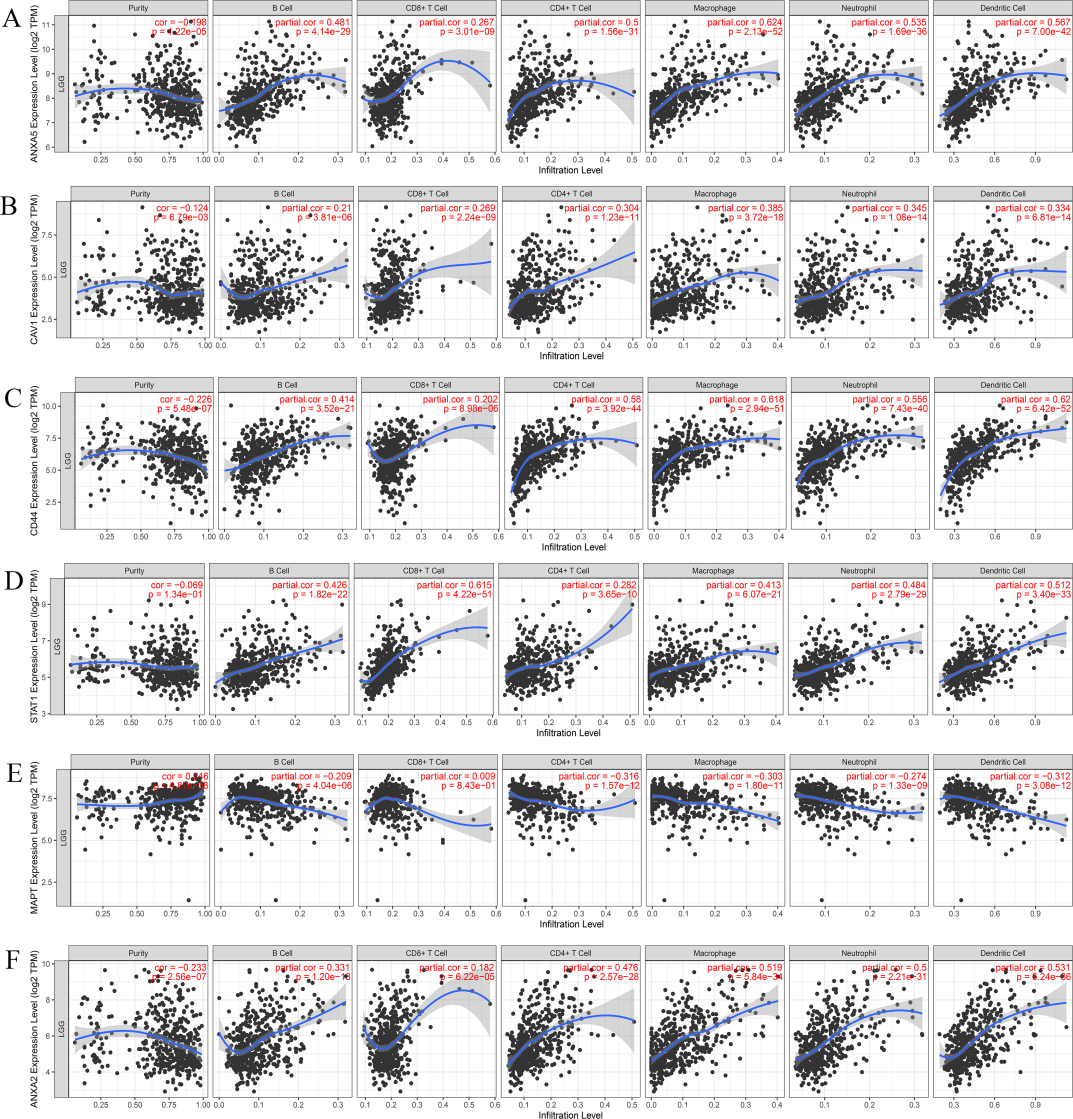
The correlation between the top 6 hub genes and immune cell in LGGs. Correlation analysis between ANXA5 **(A)**, CAV1 **(B)**, CD44 **(C)**, STAT1 **(D)**, MAPT **(E)**, as well ANXA2 **(F)** and immune cell infiltration in LGGs according to TIMER2.0 (http://timer.cistrome.org/). *P<0.05* represented statistically significant.

Next, we further investigated the correlation between the hub genes and immune cell infiltration in LGGs by using the TIMER database. The results demonstrated that there was a positive correlation between the expression of *ANXA5*, *STAT1*, *CD44*, *CAV1*, and *ANXA2*, and the infiltration of B cell, CD8^+^ T cell, CD4^+^ T cell, macrophages, neutrophils, and dendritic cells in LGGs (**Figures 7A–F**). Except for in CD8^+^ T cell, the *MAPT* expression was negatively associated with B cell, CD4^+^ T cell, macrophages, neutrophils, and dendritic cells in LGGs (**Figure 7E**). To sum up, these findings suggested that the hub genes may be also related to immune infiltration in glioma.

### Knockdown of *ANXA5* inhibited the proliferation, migration and invasion and promoted cell apoptosis of glioma cells

**Figure 8:**
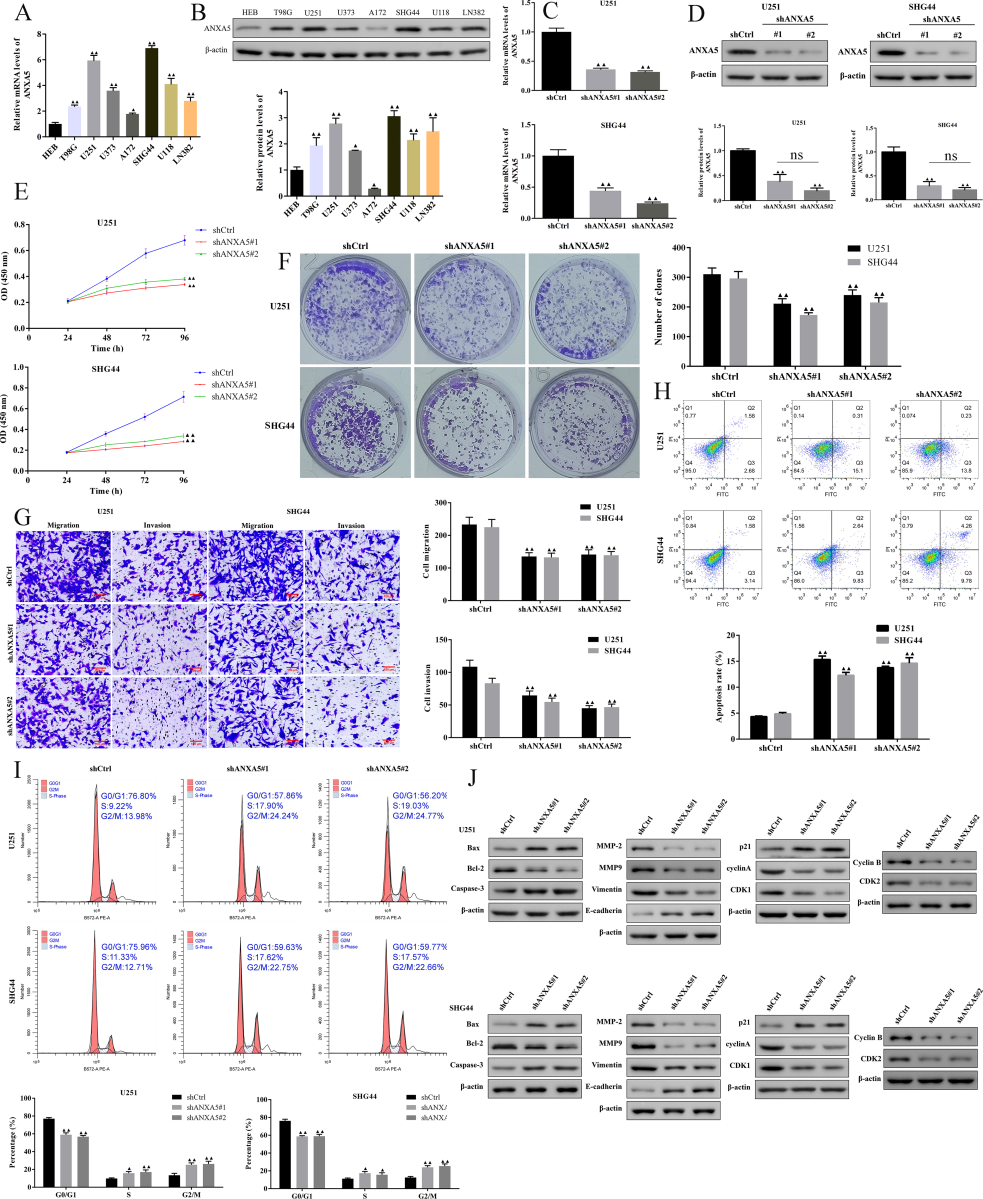
Down-regulation of ANXA5 inhibited proliferation, migration, and invasion, and promoted cell apoptosis in the U251 and SHG44 cells. The mRNA **(A)** and protein **(B)** expression levels of ANXA5 were determined by qRT-PCR and western blot, respectively. **(C-D)** Transfection efficiency of ANXA5 knock-downed shRNA in U251 and SHG44 cells was determined by qRT-PCR and western blot. **(E–I)** The effects of ANXA5 down-regulation on U251 and SHG44 cell proliferation, migration, invasion, cell apoptosis, and cell cycle were determined by CCK-8, colony formation, Transwell assays (Scale bar, 100 μm), and flow cytometry analysis, respectively. **(J)** The protein expression of Bax, Bcl-2, Caspase-3, MMP-2, MMP-9, Vimentin, E-cadherin, p21, Cyclin A, CDK1, cyclin B, and CDK2 was measured by western blot. Data were expressed as mean ± SD. ^▲^ *P < 0.05*, ^▲▲^ *P < 0.01* compared to the shCtrl group.

For the expression levels and the diagnostic value of *ANXA5*, we further investigated the effects of *ANXA5* in glioma progression. The expression levels of *ANXA5* in glioma cell lines were detected by qRT-PCR and Western blotting. As illustrated in **Figures 8A, B**, *ANXA5* expression was significantly upregulated in glioma cell lines, especially in U251 and SHG44 cells, we then selected them for further functional experimental studies. Meanwhile, we stably inhibited *ANXA5* in U251 and SHG44 cells using lentivirus plasmids. The knockdown efficiency was assessed using qRT-PCR and Western blotting, as showed a significant reduction of *ANXA5* expression in U251 and SHG44 cells (**Figures 8C, D**). Next, we investigated the effects of *ANXA5* knockdown on cell proliferation, migration, invasion, apoptosis, and cell cycle percentage in transfected U251 and SHG44 cells. As shown in **Figures 8E–H**, the knockdown of *ANXA5* dramatically inhibited cell proliferation, migration, and invasion as well as promoted cell apoptosis of glioma cells. Additionally, the results of flow cytometry demonstrated that cell cycle percentage was arrested at the S and G2/M phases in the stabilized *ANXA5* knockdown U251 and SHG44 cells (**Figure 8I**). Meanwhile, the results of western blot assays also suggested *Bax*, *Caspase-3*, *p21*, and *E-cadherin* expression were elevated while *Bcl-2*, *MMP-2*, *MMP-9*, *vimentin*, *cyclin A*, *CDK1*, *cyclin B*, and *CDK2* expression were decreased in *ANXA5*-knockdown U251 and SHG44 cells (**Figure 8J**). Taken together, these results suggested that *ANXA5* is highly expressed in glioma cells and it may contribute to the malignant characteristics of U251 and SHG44 cells.

### Knockdown of *ANXA5* inhibited tumor growth *in vivo* in glioma

**Figure 9:**
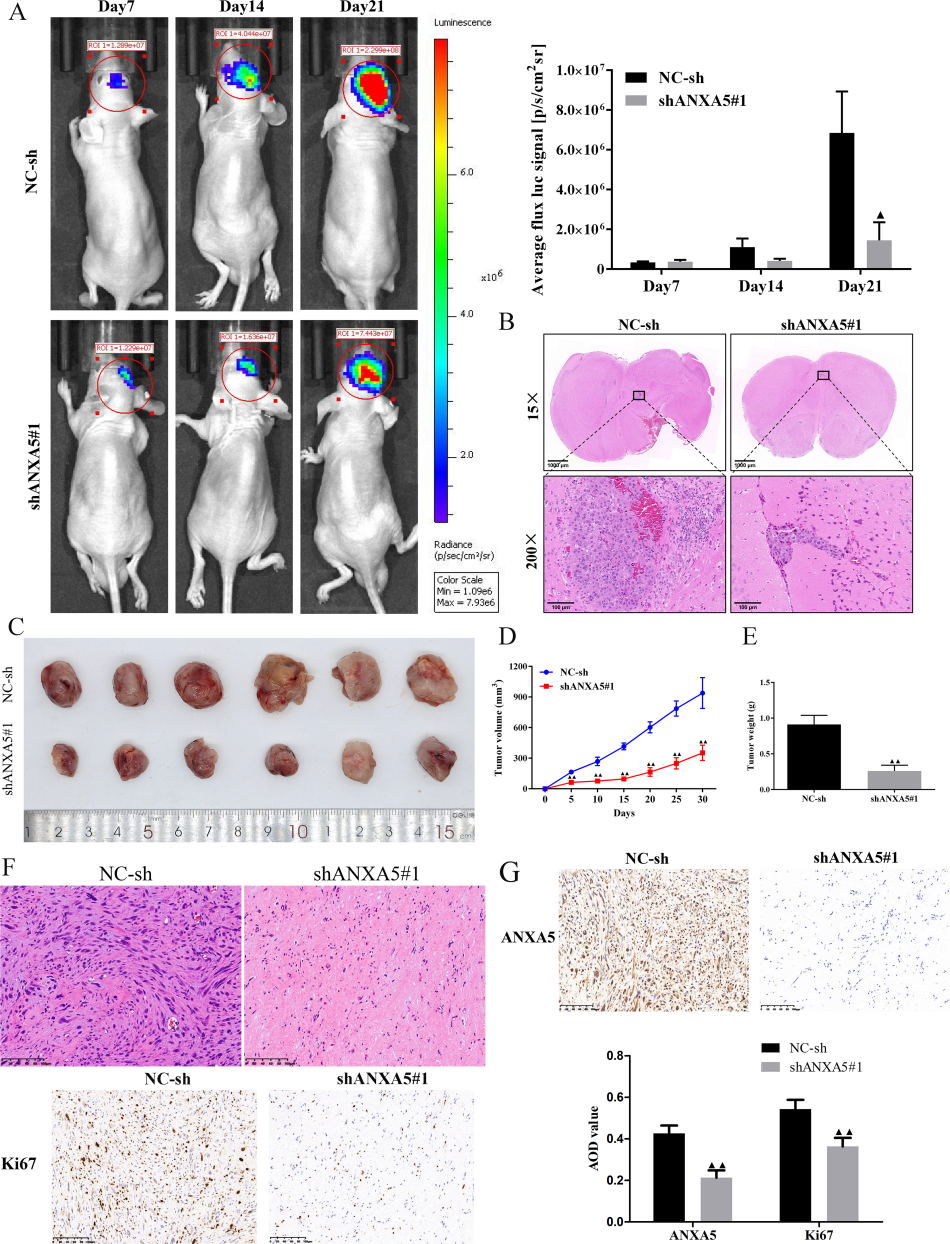
Knockdown of ANXA5 attenuated tumor growth of glioma cells *in vivo*. Luciferase-labeled U251 cells were employed to build the intracranial glioma model and tumor volume was detected *in vivo* using bioluminescent imaging. **(A)** Representative bioluminescent images and the quantification of the U251 tumor-bearing mice on days 7, 14, and 21. **(B)** HE staining analysis of tumor growth in matched primary glioma tissues. U251 cells transfected with shANXA5 or NC-sh were subcutaneously injected into the right flanks of nude mice. **(C)** Representative images of subcutaneous xenotransplanted tumor tissues. **(D-E)** The effect of ANXA5 silencing on tumor volume curve and tumor weight was recorded. **(F)** The histological change of subcutaneous tumor tissues from U251 tumor-bearing mice was evaluated by HE staining. **(G)** Representative IHC images of the subcutaneous tumor tissues from U251 tumor-bearing mice stained by ANXA5 and Ki67. Scale bar, 100 μm. All result represents the mean ± SD. ^▲^ *P < 0.05*, ^▲▲^ *P < 0.01* compared to the NC-sh group.

To further investigate the roles of *ANXA5* in U251 and SHG44 cells, we established an intracranial glioma model using NC-sh and shANXA5#1 transfected U251-luc cells as well as a xenograft model using transfected SHG44 cells. The small animal live imaging found that the knockdown of *ANXA5* significantly inhibited tumor growth *in vivo* (**Figure 9A**). The volume and weight of the tumors in the shANXA5#1 group were apparently smaller compared to the NC-sh group (**Figures 3C–E**). And, H&E staining showed that *ANXA5* knockdown crushed the compact structure of tumor tissues from intracranial and subdermal glioma models, respectively (**Figures 9B–F**). Moreover, results from the IHC assay displayed that *Ki67* levels of tumor tissues were decreased by *ANXA5* down-regulation in the xenograft model (**Figure 9G**). These results suggested that *ANXA5* knockdown restricted glioma cell growth *in vivo*.

### *ANXA5* contributes to the proliferation and migration of U251 and SHG44 cells by the *MAPK/CD44* pathway

**Figure 10:**
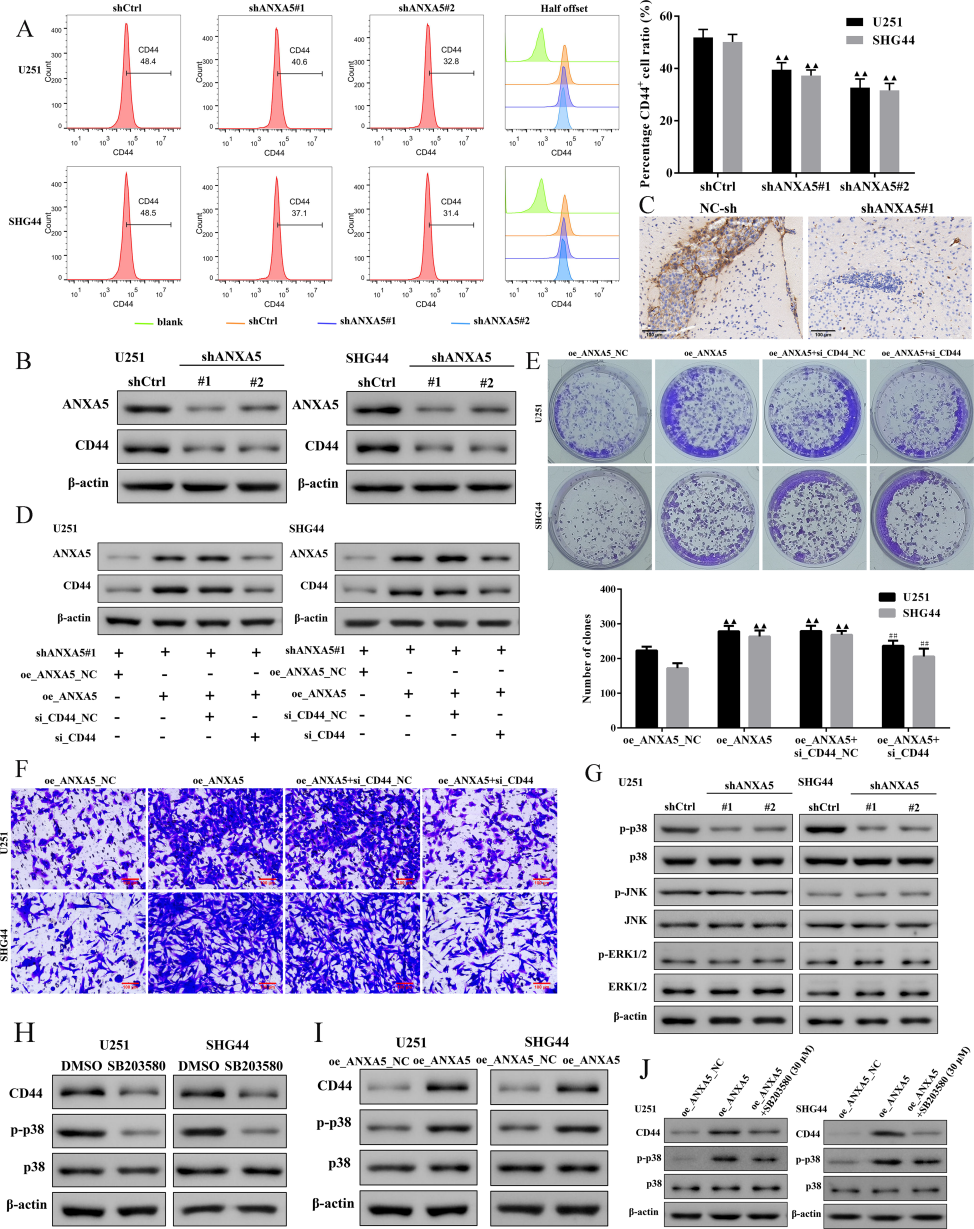
ANXA5 contributes to the proliferation and migration of glioma cells via the MAPK/CD44 pathway. **(A)** The surface expression levels of CD44 in U251 and SHG44 cells transfected with shANXA5 or shCtrl were detected by flow cytometry. **(B)** Western blot for the expression of ANXA5 and CD44 in ANXA5 knock-downed U251 and SHG44 cells. **(C)** Representative IHC images of the intracranial glioma tissue from U251 tumor-bearing mice stained by CD44. The ANXA5 stable knockdown U251 and SHG44 cells were transfected with either ANXA5 overexpression plasmid (oe_ANXA5) or normal control plasmid (oe_ANXA5_NC). The expression of CD44 was knocked down by siRNA (si_CD44) silencing. **(D)** Western blot assays of ANXA5 and CD44 protein expression in transfected cells. **(E-F)** The proliferation and migration of transfected U251 and SHG44 cells were assessed by using colony formation and Transwell assays. Scale bar, 100 μm. **(G)** Western blot for the effect of ANXA5 knockdown on the expression of p-p38, p38, p-JNK, JNK, p-ERK1/2, and ERK1/2 in transfected U251 and SHG44 cells. **(H)** The U251 and SHG44 cells were treated with a p38 inhibitor (SB203580, 30 μM) for 24h. Western blot assays of CD44, p-p38, and p38 expression in SB203580-treated U251 and SHG44 cells. **(I)** The expression levels of CD44, p-p38, and p38 in ANXA5 stable knockdown U251 and SHG44 cells with transfection of ANXA5 overexpression plasmid were analyzed using Western blots. **(J)** The expression levels of CD44, p-p38, and p38 in ANXA5-overexpressed U251 and SHG44 cells with the inhibition of p38 MAPK. Data are shown as mean ± SD. ^▲▲^ *P < 0.01* compared to shCtrl or oe_ANXA5_NC groups. ^##^ *P < 0.01* compared to oe_ANXA5+si_CD44_NC group.

The correlation analysis between *ANXA5* and *CD44* indicated that *CD44* might play an essential role in malignant behaviors involved in *ANXA5* in glioma. As shown in **Figures 10A, B**, we found that the cell surface level and protein expression levels of *CD44* were dramatically decreased in both U251 and SHG44 cells with *ANXA5* knockdown. Additionally, IHC analysis showed that the expression of *CD44* was also significantly decreased in the intracranial tumor tissues (**Figure 10C**). *ANXA5* expression was rescued by *ANXA5* overexpression plasmid in *ANXA5* stable knockdown U251 and SHG44 cells. The expression of *CD44* was inhibited by siRNA silencing (**Figure 10D**). Overexpression of *ANXA5* particularly improved the protein expression of *CD44* (**Figure 10D**). Moreover, colony formation and Transwell assays revealed that *ANXA5* restoration increased the cell proliferation and metastatic capacity in *ANXA5* stable knockdown U251 and SHG44 cells. Conversely, *ANXA5* up-regulation was unable to boost the proliferation and migration of U251 and SHG44 cells with *CD44* silencing (**Figures 10E, F**). Overall, the results pointed out that *ANXA5* might aggravate cell proliferation and migration of U251 and SHG44 cells via *CD44*. The literature indicates that *CD44* may participate in malignant behaviors in cancer partially via the *MAPK/ERK* pathway (21). In this study, results from Western blot analysis found that *ANXA5* knockdown significantly decreased the phosphorylation of p38 in U251 and SHG44 cells. Additionally, we observed that the total *p38*, *JNK*, *ERK1/2*, phosphorylated *JNK*, and phosphorylated *ERK1/2* levels in *ANXA5* stable knockdown U251 and SHG44 cells did not obviously change (**Figure 10G**). Notably, the p38 MAPK inhibitor (SB203580) significantly decreased the protein expression levels of *CD44* in U251 and SHG44 cells with inhibition of p38 phosphorylation (**Figure 10H**), indicating that p38 MAPK was the upstream that governed the *CD44* levels in glioma cells. Besides, *ANXA5* call backs promoted the protein expression levels of *CD44* and phosphorylation of p38 in *ANXA5* stable knockdown U251 and SHG44 cells (**Figure 10I**). More importantly, inhibition of *p38 MAPK* could partially mitigate the effect of *ANXA5*-overexpressed on the *CD44* expression (**Figure 10J**). Therefore, we might consider that *ANXA5* supports multifarious malignant phenotypes of glioma cells through the *MAPK/CD44* signaling pathway.

## Discussion

Gliomas pose a great challenge to the use of chemotherapeutic agents for the treatment due to limitations such as difficulty in complete resection upon anatomical features, limitations of the blood-brain barrier, and inherent apoptosis resistance of tumor cells. Even with the rapid development of 3D printing technology and the application of nanomaterials to various diseases (22–28), the molecular pathogenesis of gliomas still requires further research. In the current study, we aimed to identify critical and novel diagnostic and prognostic biomarkers or therapeutic targets for glioma. Bioinformatics methods were employed to screen DEGs between glioma and control samples to select the hub candidate targets. After pre-processing of the RNA sequencing data, 2499 DEGs from GSE209547 were identified, consisting of 765 upregulated and 1734 downregulated genes, 5372 DEGs from GSE50161 were identified, consisting of 2854 upregulated and 2518 downregulated genes, 2516 DEGs from GSE12657 were identified, consisting of 1217 upregulated and 1299 downregulated genes, and 1960 DEGs from GSE42656 were identified, consisting of 823 upregulated and 1137 downregulated genes. Then, a total of 139 overlapping DEGs were identified that could significantly participate in the occurrence and progression of glioma, suggesting they could be recognized as potential biomarkers for glioma diagnosis, prognosis, and treatment. Functional enrichment analysis of the overlapping DEGs may provide a novel understanding to clarify the mechanisms of the development of glioma. GO enrichment analysis revealed that the overlapping DEGs were mainly associated with the cell surface receptor signaling pathway, suggesting that cell surface receptors possibly play valuable roles in the progression of glioma. Furthermore, the KEGG functional enrichment analysis has shown that the overlapping DEGs were involved in the proteoglycans in cancer, *MAPK* signaling pathway, and NOD-like receptor signaling pathway. These findings could assist in illuminating the biological process of how it affected the development and outcome of gliomas.

To further examine the relationships of overlapping DEGs, the PPI network was established and revealed that *ANXA5*, *STAT1*, *CD44*, *CAV1*, *MAPT*, and *ANXA2* with a high degree of connectivity were used as hub genes. To confirm these results, we used the GEPIA and CGGA databases to examine their expression levels and prognostic value in glioma patients. For *ANXA5*, *STAT1*, *CD44*, *CAV1*, and *ANXA2*, LGG patients with high expression possessed a worse overall survival, while low expression of *MAPT* was associated with poor overall survival among patients with primary gliomas. Specially, *ANXA5* expression was positively correlated with *STAT1*, *CD44*, *CAV1*, and *ANXA2* levels as well as negatively correlated with *MAPT* repression. Notably, ROC analysis found that the prognostic signature of hub genes combined had an excellent performance in gliomas. Besides, the results of tumor microenvironment analysis suggested that *ANXA5*, *STAT1*, *CD44*, *CAV1*, and *ANXA2* expression was positively correlated with the infiltration of B cell, CD8^+^ T cell, CD4^+^ T cell, macrophages, neutrophils, and dendritic cells. Altogether, these findings suggested that *ANXA5*, *STAT1*, *CD44*, *CAV1*, *MAPT*, and *ANXA2* might play a vital role in the pathogenesis of gliomas.

Meanwhile, it has been found that *ANXA5* and *ANXA2* could be used as independent prognostic biomarkers suggesting undesirable outcomes in glioblastoma (29). Additionally, *ANXA5* and *ANXA2* expressions were upregulated in advanced hepatocellular carcinoma stages which were proposed as potentially useful biomarkers for poor survival in liver cancer patients (30). *STAT1*, a cytoplasmic transcription factor, exerts significant effects in maintaining cell growth and migration in glioblastoma (31). However, a study by Hua et al. showed that overexpression of *STAT1* inhibited the proliferation of U251 and U87 cells (32). *CD44*, a multifunctional molecule, could not only promote the migration and invasion of glioblastoma cells under severe hypoxia (33) but also serve as a prognostic biomarker in glioma patients with a poor prognosis (34). *CAV1*, a principal structural protein of caveolae, was found to be an important contributor to the tumorigenicity of glioblastoma cells (35–37), and also could serve as an independent prognostic marker in glioblastoma (38). Similarly, Zaman et al. illustrated that high expression of *MAPT* is significantly associated with increased overall and disease-free survival in LGG patients (39). Additionally, Nakata et al. have reported that high *MAPT* expression is not only related to IDH mutation status but also may aid in the prevention of glioma by disrupting tumor invasion (40). Hence, the mechanism of how *ANXA5*, *STAT1*, *CD44*, *CAV1*, *MAPT*, and *ANXA2* contributed to the gliomas still need further research.

This study estimated the tumor-promoting role of *ANXA5* in transfected U251 and SHG44 cells and also elucidated its underlying mechanism. The results showed that the expression levels of *ANXA5* were higher in glioma cell lines compared with the human normal glial cell line HEB. Importantly, CCK-8, colony formation, and flow cytometric assays consistently showed that *ANXA5* knock-down inhibited glioma cell proliferation and altered the cell cycle distribution. Furthermore, Transwell assays found that down-regulation of *ANXA5* inhibited migration and invasion in U251 and SHG44 cells. Additionally, *ANXA5* knockdown significantly inhibited the tumor growth *in vivo*, suggesting that it could be a promising therapeutic target in gliomas. Among the hub genes, we also found that the expression of *CD44* was decreased by *ANXA5* silencing in U251 and SHG44 cells. Meanwhile, we also found that *ANXA5* promoted the expression of *CD44* by activating the *p38 MAPK* pathway, and the knockdown of *CD44* in *ANXA5*-recovered U251 and SHG44 cells could inhibit the effects of *ANXA5*. Hence, it is reasonable to conclude that *ANXA5* promotes the proliferation and migration of glioma cells by the *MAPK/CD44* pathway. Thus, we believe that *ANXA5* is an important pathogenic factor in glioma. However, there were some limitations in this study. First, the results of the bioinformatic analysis may be inconsistent due to differences in public databases. Second, the roles and underlying mechanisms of all hub genes were not fully studied. Moreover, the validation of *ANXA5* expression in large tumor samples needs to be further studied. Therefore, further molecular and biological experiments are needed to confirm the roles of the hub genes in glioma.

## Conclusions

In summary, a systematic bioinformatics analysis of DEGs demonstrated that *ANXA5*, *STAT1*, *CD44*, *CAV1*, *MAPT*, and *ANXA2* might serve as potential biomarkers and therapeutic targets for glioma. Moreover, we found *ANXA5* was highly expressed in glioma cells and contributes to tumor progression *in vitro* and *in vivo* and illustrated the potential molecular mechanisms that *ANXA5* functions through the *MAPK/CD44* pathway, which might provide a basis of clinical significance for *ANXA5* in glioma treatment.

## Data Availability

The original contributions presented in the study are included in the article/supplementary material. Further inquiries can be directed to the corresponding author/s.
